# Supplementary material for: Efficacy of Organ Preservation Strategy by Adjuvant Chemoradiotherapy after Non-Curative Endoscopic Resection for Superficial SCC: A Multicenter Western Study
Source: Cancers (Basel). 2023 Jan 18;15(3):590. doi: 10.3390/cancers15030590 (PMC9913357; doi:10.3390/cancers15030590)
Supplement: Supplementary file 1 [file cancers-15-00590-s001.zip › cancers-2072830-supplementary.pdf]

cancers MDPI

S1

Center and period bias assessment

The endoscopic centers were divided into three groups (only EMR, only ESD, or EMR and ESD). The grouping was not an independent predictor of recurrence-free survival (P = 0.316). The recurrence-free survival rate within the third tertile of the inclusion period was similar in each tertile period (P = 0.284) **(Supplementary figure 1).**

Complications after endoscopic resection

The overall complication rate was 8% and was similar in the ESD (12.1%) and EMR (4.2%) groups (P = 0.089). Intraoperative perforations occurred in six procedures (one EMR, five ESD). In all cases the treatment was conservative, using a self-expandable metallic stent and antibiotics. Delayed bleeding occurred in 1.5% of procedures (2/137). During follow-up, the rate of postoperative strictures was 17.5% (24/137). Patients with strictures were successfully treated by endoscopy, and any patient with a refractory stricture required secondary surgery.

Local recurrence

The rate of local recurrence was 21.1% in the EMR group and 3% in the ESD group and this difference was statistically significant (P = 0.016) (Supplementary Table 2 and 3). In univariable analyses resection by EMR (P = 0.016), piecemeal resection (P = 0.012), and non-tumor-free lateral margin (P < 0.001) were associated with local recurrence. However, in the multivariable analysis no factor was significantly associated with local recurrence (Supplementary Table 3).

S2

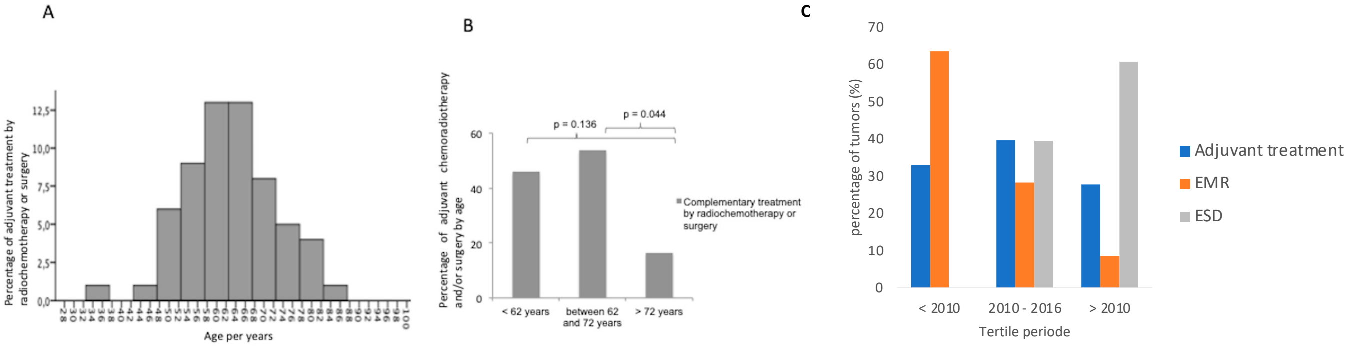

Supplementary Figure S1. Adjuvant chemoradiotherapy and/or surgery by age. Panel A: Percentage of adjuvant chemoradiotherapy and/or surgery by age; Panel B: Comparison of rates of adjuvant chemoradiotherapy and/or surgery by age group (< 62 years; between 62 and 72 years; and > 72 years). Panel C: Comparison of rates of adjuvant chemoradiotherapy, and endoscopic resection by EMR or ESD.

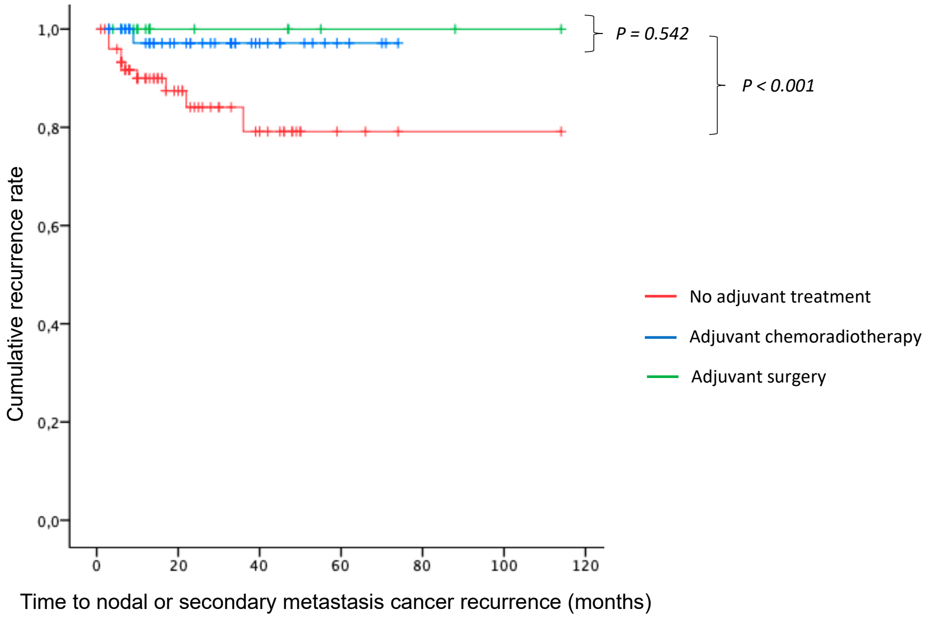

Supplementary Figure S2. Nodal or metastatic recurrence-free survival rates in patients with tumors invading the muscularis mucosae (pT1a-m3) or the submucosa (pT1b-Sm) with and without adjuvant chemoradiotherapy and/or surgery.

Supplementary Table S1. Number of EMR and ESD procedures performed per center.

Centre, n(%)	ESD group (n=66)	EMR group (n=71)
1	5(7.6)	6(8.5)
2	11(16.7)	0(0.0)
3	0(0.0)	8(11.3)
4	0(0.0)	10(14.1)
5	16(24.2)	1(1.4)
6	9(13.6)	0(0.0)
7	3(4.5)	0(0.0)
8	0(0.0)	11(15.5)
9	3(4.5)	34(47.9)
10	17(25.8)	0(0.0)
11	2(3.0)	1(1.4)

ESD, endoscopic submucosal dissection; EMR, endoscopic mucosal resection.

Supplementary Table S2. Comparisons of the EMR and ESD groups were performed in intention-to-treat, choice between EMR or ESD was decided before to start the endoscopic resection.

	EMR group (n = 71)	ESD group (n = 66)	*P*-value*
Male n (%)	57 (80.3)	46 (69.7)	0.152
Mean age (years, (range))	63.6 (35-87)	64.1 (44-90)	0.189
Median size (mm, (range))	15.0 (3.0-30.0)	35.7 (8.0-90.0)	<0.001
Tumor circumference of the esophageal lumen > 1/3 n (%)	34 (47.9)	50 (75.8)	0.001
Piecemeal resection n (%)	48 (67.6)	2 (3.0)	<0.001
Lateral margin-free cancer (%) (R0; R1; Rx) [a]	(14.1; 18.3; 67.6)	(90.9; 6.1; 3.0)	<0.001
Tumor infiltration depth m3-sm1 n (%)	48 (67.6)	43 (65.2)	0.761

Adjuvant treatment by chemoradiotherapy or/and surgery n (%)	30 (42.3)	31 (47.0)	0.579
Mean follow-up (months, (range))	41.4 (1-135)	17.6 (3-55)	<0.001

ESD, endoscopic submucosal dissection; EMR, endoscopic mucosal resection; m3, cancer invading the muscularis mucosae, sm1: cancer invading the submucosa. *Univariate analyses were carried out by chi-squared test for qualitative variables. For tumor size and age, [a] univariate t-test was used. a Lateral margins were considered R0 for tumor-free margins, R1 for non-tumor-free margins and Rx when the pathology was not evaluable, for example due to piecemeal resection.

Supplementary Table S3. Results according to local cancer recurrence during the follow-up.

	Local cancer recurrence		Univariate analysis
	Yes (n =17)	No (n =120)	*P*-value[1]
Male n (%)	12 (70.6)	91 (75.8)	0.407
Mean age, (years, (range))	65.7 (51-78)	63.6 (35-90)	0.074
Median tumor size, (mm, (range))	20.0 (10.0-40.0)	25.0 (3.0-90.0)	0.210
Tumor circumference of the esophageal lumen > 1/3 n (%)	8 (47.1)	76 (63.3)	0.278
EMR resection n (%)	15 (88.2)	56 (46.7)	0.016
Piecemeal resection n (%)	12 (70.6)	38 (31.7)	0.012
Tumor infiltration depth > Sm1 n (%)	6 (35.3)	40 (33.3)	0.854
Differentiation G2 and G3‡, n (%)	4 (23.5)	38 (31.7)	0.696
Lymphovascular invasion, n (%)	1 (5.9)	18 (15.0)	0.397
R1 or Rx [a] lateral margins n (%)	17 (100.0)	50 (24.2)	<0.001
Adjuvant treatment by chemoradiotherapy n (%)	5 (29.4)	56 (46.7)	0.126
Median follow-up (months, (range))	46.0 (9-135)	20.5 (1-115)	0.074

CI, confidence interval; EMR, endoscopic mucosal resection; m3, cancer invading the muscularis mucosae, sm, cancer invading the submucosa; R1, margin not tumor free; SCC, squamous cell carcinoma. [a] R1 for non-tumor-free margins and Rx when the pathology was not evaluable, for example, due to piecemeal resection. [1] Univariate analyses were carried out using the log-rank test for qualitative variables. For age and tumor size, a univariate Cox model was used. All significant factors, together with those of borderline significance ($P < 0.2$), were included in the multivariate analysis, which used the Cox proportional hazards model. In the multivariate models, only variables with a $P < 0.05$ were retained.

Supplementary Table S4. Characteristics of adjuvant chemoradiotherapy and/or surgery.

	Complementary treatment		Univariate analysis
	Chemoradiotherapy (n = 46)	surgery (n = 15)	*P* value*
Male, n (%)	36 (78.3)	11 (73.3)	0.730
Age, mean (range), years	63.8 (35-83)	60.0 (44-81)	0.190
ASA score ≥3, n (%)	24 (52.2)	8 (53.3)	0.938

Tumor size, median (range), mm	25 (5-65)	30 (10-81.7)	0.022
EMR resection, n (%)	24 (52.2)	6 (40.0)	0.413
Piece-meal resection, n(%)	15 (32.6)	4 (26.7)	0.757
Tumor infiltration depth m3–sm1, n (%)	27 (58.7)	7 (46.7)	0.415
Differentiation G2 and G3‡, n (%)	17 (37.0)	5 (33.3)	0.800
Lymphovascular invasion, n (%)	9 (19.6)	2 (13.3)	0.716
Local recurrence cancer, n (%)	5 (10.9)	0 (0.0)	0.321

ASA, American Society of Anesthesiologists; EMR, endoscopic mucosal resection; m3, invasive carcinoma limited to the muscularis mucosae; sm, invasive carcinoma limited to the submucosa. *Univariate analyses were carried out by chi-squared test for qualitative variables. For age and tumor size, a univariate t-test was used. For local recurrence, a univariate Cox model was used. ‡G2 was moderately differentiated and G3 was poorly differentiated.

Supplementary Table S5. Characteristics of adjuvant chemoradiotherapy.

	Adjuvant treatment by chemoradiotherapy		Univariate analysis
	Yes (n = 46)	No (n = 76)	*P* value*
Male, n (%)	36 (78.3)	56 (73.7)	0.570
Age, mean (range), years	63.8 (35-83)	64.7 (44-90)	0.886
ASA score ≥3, n (%)	24 (52.2)	39 (51.3)	0.927
History of head-and-neck cancer n (%)	19 (41.3)	28 (36.8)	0.624
Tumor size, median (range), mm	25 (5-65)	20 (3-90)	0.861
EMR resection, n (%)	24 (52.2)	6 (40.0)	0.413
Piece-meal resection, n(%)	15 (32.6)	31 (40.8)	0.366
Tumor infiltration depth m3–sm1, n (%)	27 (58.7)	57 (75.0)	0.059
Differentiation G2 and G3‡, n (%)	17 (37.0)	20 (26.3)	0.215
Lymphovascular invasion, n (%)	9 (19.6)	8 (10.5)	0.162

ASA, American Society of Anesthesiologists; EMR, endoscopic mucosal resection; m3, invasive carcinoma limited to the muscularis mucosae; sm, invasive carcinoma limited to the submucosa. *Univariate analyses were carried out by chi-squared test for qualitative variables. For age and tumor size, a univariate t-test was used. ‡G2 was moderately differentiated and G3 was poorly differentiated.

Supplementary Table S6. Risk factors associated with nodal or distal metastasis recurrence during.

	Nodal or secondary metastasis cancer recurrence	Univariate analysis	Multivariate analysis

	Yes (n =11)	No (n =111)	*P*-value[1]	Hazard ratio (95% CI)
Male n (%)	8 (75.7)	84 (72.7)	1.000	
Mean age, (years, (range))	70.2 (56-83)	63.8 (35-90)	0.030	1.083(1.015-1.156)
History of head-and-neck cancer n (%)	4 (36.4)	43 (38.7)	1.000	
Median tumor size, (mm, (range))	20.0 (7.0-71.5)	25.0 (3.0-90.0)	0.478	
Tumor circumference of the esophageal lumen > 1/3 n (%)	4 (36.4)	69 (62.2)	0.115	
EMR resection n (%)	7 (63.6)	58 (52.3)	0.470	
Piecemeal resection n (%)	6 (54.5)	40 (36.0)	0.328	
R1 or Rx [a] lateral margins, n (%)	6 (54.5)	54 (48.6)	0.709	
Rx[a]depth margins, n (%)	3 (27.3)	18 (16.2)	0.400	
Tumor infiltration depth > Sm1 n (%)	6 (54.5)	32 (28.8)	0.095	4.446(1.286-15.515)
Differentiation G2 and G3[‡], n (%)	6 (54.5)	31 (30.1)	0.171	
Lymphovascular invasion, n (%)	1 (9.1)	16 (15.8)	1.000	
No adjuvant treatment by chemoradiotherapy n (%)	1 (9.1)	45 (40.5)	0.051	8.072(1.006-64.802)
Median follow-up (months, (range))	34.0 (9-112)	23.0 (1-135)	0.180	

CI, confidence interval; EMR, endoscopic mucosal resection; sm1, cancer invading the submucosa; R1, margin not tumor free. [a] R1 for microscopic margins positive for tumor and Rx when the pathology was not evaluable, for example due to piecemeal resection and/or coagulation artifacts. [1] Univariate analyses were carried out by log-rank test for qualitative variables. For age and tumor size, a univariate Cox model was used. All significant factors, together with those of borderline significance ($P < 0.2$), were included in the multivariate analyses, which used the Cox proportional hazards model. In the multivariate models, only variables with a $P < 0.05$ were included.
